# Disseminated Tuberculosis and Chronic Mucocutaneous Candidiasis in a Patient with a Gain-of-Function Mutation in Signal Transduction and Activator of Transcription 1

**DOI:** 10.3389/fimmu.2017.01651

**Published:** 2017-12-06

**Authors:** Sigifredo Pedraza-Sánchez, Jose Luis Lezana-Fernández, Yolanda Gonzalez, Luis Martínez-Robles, María Laura Ventura-Ayala, Stanislaw Sadowinski-Pine, Margarita Nava-Frías, Sarbelio Moreno-Espinosa, Jean-Laurent Casanova, Anne Puel, Stephanie Boisson-Dupuis, Martha Torres

**Affiliations:** ^1^Unidad de Bioquímica, Instituto Nacional de Ciencias Médicas y Nutrición Salvador Zubirán, México City, México; ^2^Laboratorio de Fisiología Pulmonar, Hospital Infantil de México, México City, México; ^3^Departamento de Investigación en Microbiología, Instituto Nacional de Enfermedades Respiratorias, México City, México; ^4^Departamento de Patología, Hospital Infantil de México, México City, México; ^5^Departamento de Infectología, Hospital Infantil de México, México City, México; ^6^St Giles Laboratory of Human Genetics of Infectious Diseases, Rockefeller Branch, Rockefeller University, New York, NY, United States; ^7^Laboratory of Human Genetics of Infectious Diseases, Necker Branch, INSERM UMR 1163, Paris, France; ^8^Imagine Institute, Paris Descartes University, Paris, France

**Keywords:** signal transduction and activator of transcription 1, gain-of-function mutation, mycobacterial infection, tuberculosis, immunodeficiency, IL-17, interferon gamma

## Abstract

In humans, recessive loss-of-function mutations in *STAT1* are associated with mycobacterial and viral infections, whereas gain-of-function (GOF) mutations in *STAT1* are associated with a type of primary immunodeficiency related mainly, but not exclusively, to chronic mucocutaneous candidiasis (CMC). We studied and established a molecular diagnosis in a pediatric patient with mycobacterial infections, associated with CMC. The patient, daughter of a non-consanguineous mestizo Mexican family, had axillary adenitis secondary to BCG vaccination and was cured with resection of the abscess at 1-year old. At the age of 4 years, she had a supraclavicular abscess with acid-fast-staining bacilli identified in the soft tissue and bone, with clinical signs of disseminated infection and a positive Gene-X-pert test, which responded to anti-mycobacterial drugs. Laboratory tests of the IL-12/interferon gamma (IFN-γ) circuit showed a higher production of IL-12p70 in the whole blood from the patient compared to healthy controls, when stimulated with BCG and BCG + IFN-γ. The whole blood of the patient produced 35% less IFN-γ compared to controls assessed by ELISA and flow cytometry, but IL-17 producing T cells from patient were almost absent in PBMC stimulated with PMA plus ionomycin. Signal transduction and activator of transcription 1 (STAT1) was hyperphosphorylated at tyrosine 701 in response to IFN-γ and -α, as demonstrated by flow cytometry and Western blotting in fresh blood mononuclear cells and in Epstein-Barr virus lymphoblastoid cell lines (EBV-LCLs); phosphorylation of STAT1 in EBV-LCLs from the patient was resistant to inhibition by staurosporine but sensitive to ruxolitinib, a Jak phosphorylation inhibitor. Genomic DNA sequencing showed a *de novo* mutation in *STAT1* in cells from the patient, absent in her parents and brother; a known T385M missense mutation in the DNA-binding domain of the transcription factor was identified, and it is a GOF mutation. Therefore, GOF mutations in *STAT1* can induce susceptibility not only to fungal but also to mycobacterial infections by mechanisms to be determined.

## Introduction

**Figure 1:**
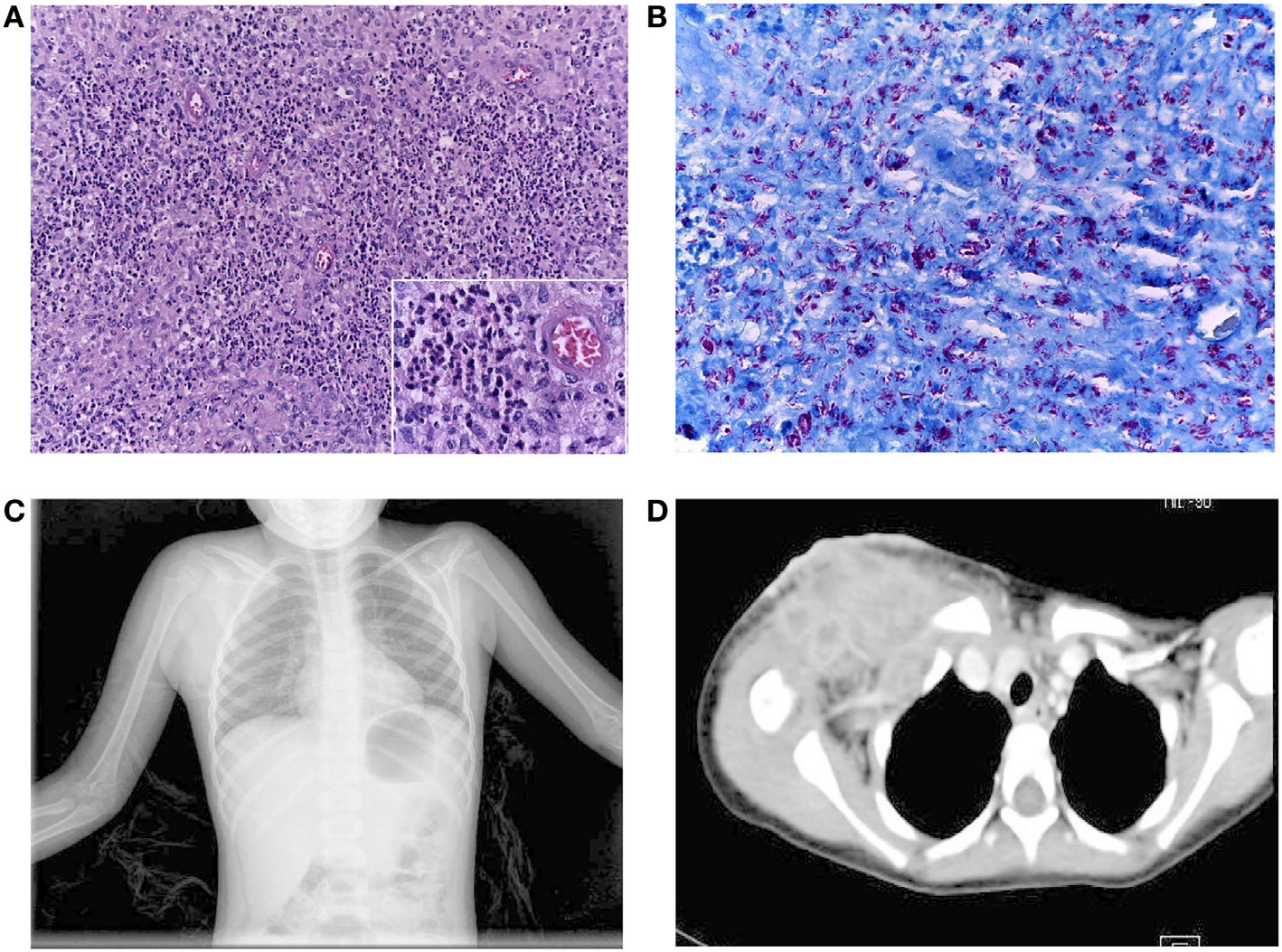
**(A)** Inflammatory reaction in the soft clavicular tissue was composed predominantly of numerous polymorphonuclear neutrophils and groups of epithelioid cells, (insert), without giant cells. H&E staining, 200× magnification. **(B)** AFS showing the abundant density of acid-fast bacilli in the same tissue. AFS, 400× magnification. **(C)** Chest X-rays showing an increase in soft tissue in the right supraclavicular region. **(D)** Contrast mediastinum CT showing the presence of multiple abscesses (lymphatic nodes with hypodense centers extending to the axillary region). There was no mediastinal invasion.

Our patient is a 9-year-old girl, who was born as a second child to young healthy non-consanguineous parents, from Oaxaca, Mexico, without a family history of recurrent infections. She received the BCG vaccine during the first month of life, and at the age of 1 year, she developed an axillary adenitis at the site of the BCG vaccination and subsequent abscess, which was debrided with relief of symptoms. She was admitted to the hospital twice with symptoms of lower airways infection at the age of 1 and 3 years with no microorganism determination. During the second hospitalization, acid-fast bacilli (AFB) were reported in a sample of gastric juice; mycobacterial cultures were negative, a PPD test was also negative, and she did not receive any specific treatment. The patient presented also with recurrent episodes of oral candidiasis starting at the age of 8 months, and she received multiple drug treatments without achieving its complete eradication. At four and a half years old, the patient had infra-clavicular swelling with rubor and pain; she was diagnosed with a soft tissue abscess, and she was treated with Ceftriaxone, Amikacin, Clarithromycin, and Trimethroprim/Sulfamethoxazole. However, the abscess volume increased reaching 10 cm × 10 cm with undefined limits and skin erosion; the patient had an intermittent low-grade fever (38.0–38.5°C) with one peak each day for the next 3 months, and she received several courses of antibiotic without improvement. Then, the patient was admitted to the hospital because general illness, including fever, and tuberculosis infection was suspected. After hospital admission, an excisional clavicular biopsy reported inflammation and abundant AFB (10 bacilli per field, in 20 fields) in the soft tissue (Figures 1A,B) and osteomyelitis (Figure 1C). Computerized axial tomography showed evidence of multiple infectious abscesses (Figure 1D). PCR analysis of material from the supraclavicular biopsy was positive for *Mycobacterium tuberculosis* complex and the Gene-X-pert test was positive for *M. tuberculosis* sensitive to rifampin. A diagnosis of disseminated tuberculosis was made, and the patient received anti-mycobacterial treatment with Rifampin, Isoniazid, Pirazynamide, and Ethambutol at conventional doses. A repeat biopsy of supraclavicular abscess showed nine AFB in 100 fields; with this improvement, the patient was discharged from the hospital on continued anti-mycobacterial treatment with Rifampin plus Isoniazid for18 months, with good clinical evolution.

The patient had neutropenia and lymphopenia during infection episodes; serum IgA levels were transiently low in several assessments, returning to normal values after recovering from active infections. Values for IgG, IgM, and IgE were normal. The patient was diagnosed with chronic hepatitis, with high values of alanine-aminotransferase, aspartate-aminotransferase, and gamma-glutamyltranspeptidase, most likely due to anti-mycobacterial and anti-fungal treatments, since a liver biopsy showed mild chronic hepatitis, without fibrosis or copper deposits. Additional tests for liver function were normal. Serology tests for viral infections (including hepatitis A, B, and C, CMV, HIV, and EBV) were all negatives. Tests for autoantibodies against DNA, cardiolipin, beta-2 glycoprotein, endomysium, and smooth muscle were all negatives.

**Figure 2:**
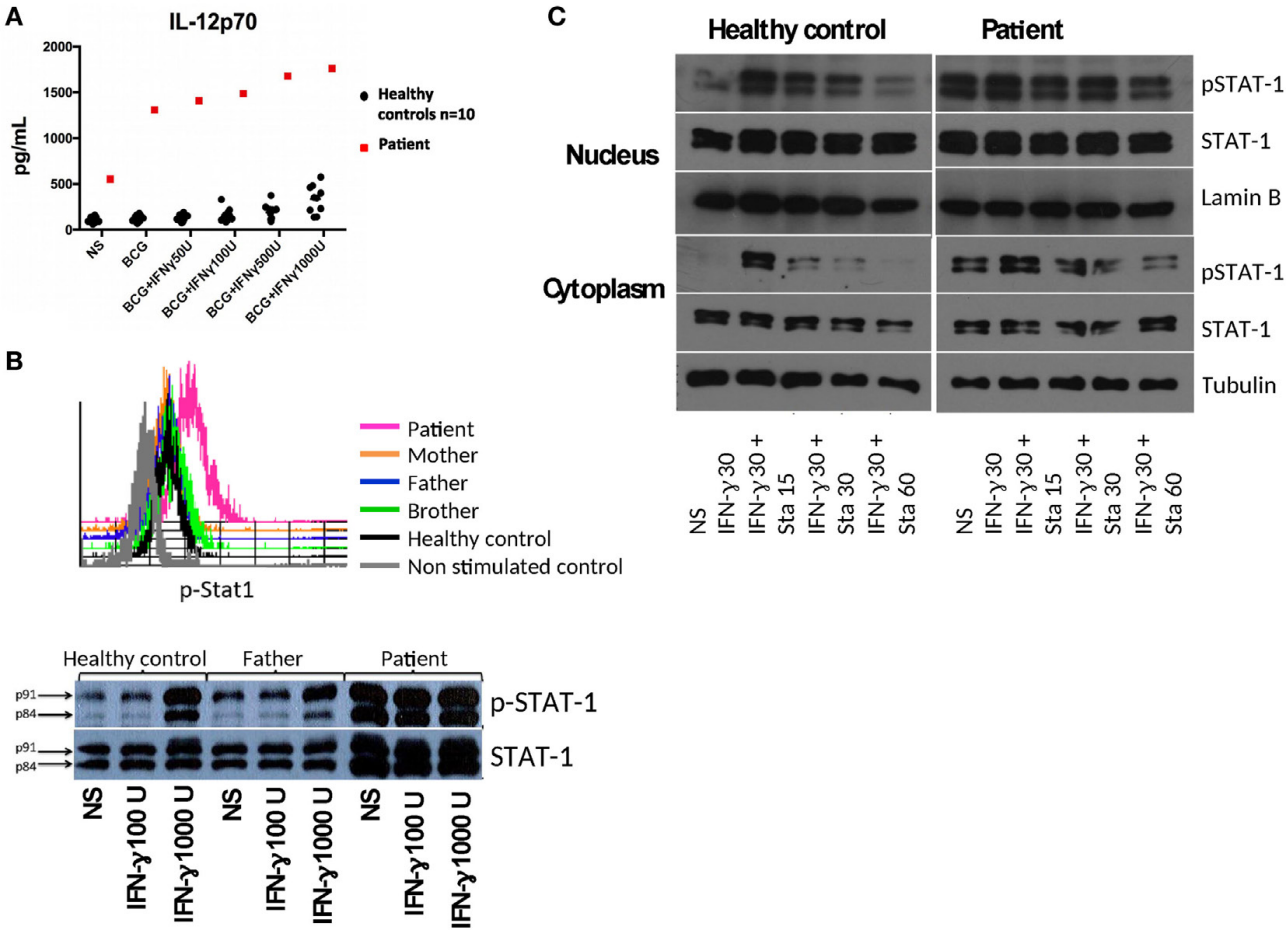
**(A)** IL-12p70 production in diluted whole blood from the patient and controls stimulated with BCG without or with increasing doses of interferon gamma (IFN-γ). **(B)** Phospho-signal transduction and activator of transcription 1 (STAT1) levels assessed in IFN-γ stimulated mononuclear cells (selected CD14+ monocytes) by flow cytometry and by Western blot (WB) in Epstein-Barr virus lymphoblastoid cell lines (EBV-LCLs). **(C)** Control and patient EBV-LCLs were stimulated with IFN-γ and incubated with Staurosporine to assess p-STAT1 levels by WB. Ten micrograms of protein of either cytoplasmic or nuclear extracts for each condition were separated by SDS-PAGE and electrotransferred to PDVF membranes. WBs were performed with anti-p-STAT1, anti-STAT1, and anti-tubulin (anti-lamin B for nuclear extracts) antibodies, with stripping steps between each antibody. WB films were scanned and the strips corresponding to each molecule (p-STAT1, STAT1, tubulin, or lamin B) were trimmed to compose the figure; the same brightness and contrast were utilized for each strip. The scans of the original WBs are included in the Supplementary material.

We found a relatively low production of interferon gamma (IFN-γ) in response to BCG and BCG + IL-12 treatment of diluted whole blood in the patient compared to healthy controls (BCG = 891 pg/mL vs BCG + IL-12 = 5,025 pg/mL for patient, compared to nine healthy controls: GeoMean ± SEM with BCG = 1,369 ± 1,878 and with BCG + IL-12 = 9,579 ± 1,935 pg/mL). The IL-12Rβ1 expression levels on PHA-T cell blasts by flow cytometry were normal in the patient (data not shown). *In vitro* responses to IFN-γ showed an increased production of IL-12p70 in the patient compared to healthy controls (Figure 2A), upon BCG and BCG + IFN-γ stimulation, suggesting an alteration in the IFN-γ receptor or downstream signaling. Membrane expression of IFN-γ receptor 1 (CD119) on patient’s CD14+ cells was similar to healthy controls (data not shown).

Signal transduction and activator of transcription 1 (STAT1) showed an increased tyrosine phosphorylation at position 701 in CD14+ cells in PBMCs from the patient stimulated with IFN-γ compared to controls, as assessed by flow cytometry (Figure 2B, histograms); similar STAT1 hyperphosphorylation was found in Epstein-Barr virus lymphoblastoid cell lines (EBV-LCLs) from the patient (Figure 2B, Western blot), suggesting a gain-of-function (GOF) mutation in *STAT1*. Since previous studies demonstrated the involvement of impaired dephosphorylation for other GOF mutations in *STAT1* (1–3), we characterized the inhibition of phosphorylation by staurosporine in EVB-LCLs. We found resistance to staurosporine-mediated inhibition of phosphorylation of STAT1 in the patient than in healthy control (Figure 2C).

**Figure 3:**
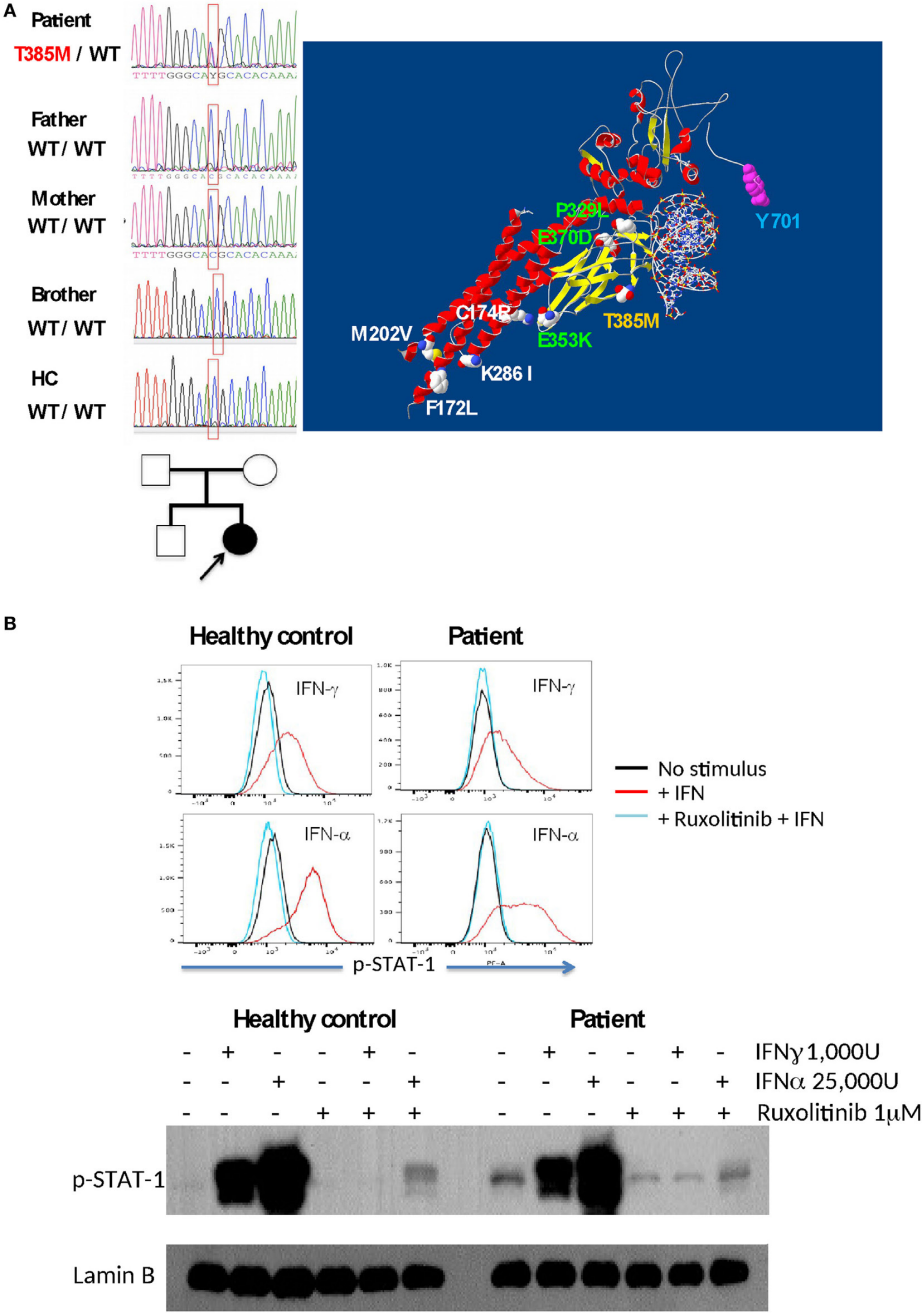
**(A)** The T385M mutation was found to be as a *de novo* mutation in the patient (DNA sequences and familial tree). The T385M mutation in the DNA-binding domain is shown in yellow in a signal transduction and activator of transcription 1 (STAT1)/DNA model; other gain-of-function mutations associated with fungal infections are shown in green (DNA-binding domain) and white (C–C-domain), and p-Tyrosine 701 is labeled with blue. **(B)** Inhibition of interferon-induced STAT1 phosphorilation in EBV-LCL by Ruxolitinib in a healthy control and in the patient, as demonstrated by intracellular flow cytometry (upper panel) and Western blot (lower panel).

Sequencing of the *STAT1* gene in PBMCs showed a mutation in the DNA-binding domain (c.1154 C>T, p.T385M) only in the patient (absent in the parents and brother), indicating a *de novo* mutation (Figure 3A). Threonine 385 is a highly conserved amino acid in several vertebrate species, representing a critical amino acid for STAT1 function; this mutation has already been shown to cause STAT1 GOF (4). Recently, the use of the Jak kinase inhibitor Ruxolitinib was demonstrated to correct STAT1 hyperphosphorylation and ameliorate Candida infection (5, 6); therefore, we tested and demonstrated inhibition of STAT1 phosphorylation by Ruxolitinib in EBV-LCLs from our patient upon stimulation with IFN-α and IFN-γ (Figure 3B).

## Background

Disseminated mycobacterial infections that occur in children are usually associated with primary immunodeficiency and are frequently related to impaired IFN-γ-dependent immune responses. Children affected with the Mendelian susceptibility to mycobacterial diseases show variable susceptibility to mycobacterial infections ranging from weakly virulent species, such as *Mycobacterium bovis* BCG and *Mycobacterium avium*, to the more virulent *M. tuberculosis*; these patients have mutations in genes controlling the IFN-γ production (*IL12B, IL12RB1, IRF8, ISG15*, and *NEMO*) or in genes involved in the response to IFN-γ (*IFNGR1, IFNGR2, STAT1, IRF8*, and *CYBB*) (7). These genes encode distinct proteins participating in the IFN-γ/IL-12 circuit, which is crucial for the immune response against mycobacteria in humans (7–10).

Engagement of IFN-γ by its receptor in macrophages activates JAK1 and JAK2 intracellular kinases, which auto- and trans-phosphorylate themselves. They phosphorylate tyrosine (Y) 440 on the IFN-γ receptor 1 creating docking sites for STAT1 coupling, which in turn is phosphorylated at Tyrosine 701 (pY701) by the same JAKs. Once STAT1 is phosphorylated, it forms homodimers in the cytoplasm; these homodimers are called GAFs or Gamma-activated factors, which translocate to the nucleus where they bind to specific DNA sequences called GASs or Gamma-activated sequences. GAFs are responsible for IFN-γ-dependent gene induction, enabling the macrophage to perform intracellular killing of phagocytized microbes (10, 11). STAT1 also forms heterodimers with STAT2 upon IFN-α or IFN-β activation mediated by their respective receptors; these STAT1/STAT2 heterodimers bind to IRF9 to form the mature transcription factor ISGF3, which binds to the specific DNA sequence ISRE and induces transcription of genes that participate in the antiviral immune response (12).

Mutations in *STAT1* in humans have previously been identified. Recessive loss-of-function (LOF) mutations in *STAT1* cause reduced signaling involving the STAT1 transcription factor, affecting immunity to intracellular pathogens primarily, like mycobacteria and viral infections due to impaired IFN-γ and IFN-α signaling, respectively (13). Dominant LOF mutations in STAT1 impair its phosphorylation or DNA-binding activity and are associated with mycobacterial disease due to a specific impairment in IFN-γ signaling (14). In contrast, GOF mutations in *STAT1* cause an increased phosphorylation of Y701 of STAT1 and a reduced production of IL-17 by T cells from affected persons; these mutations have been found in patients with fungal diseases, primarily caused by *Candida* spp. as well as by other fungal diseases such as Coccidioidomicosis and Histoplasmosis (1, 15, 16). Chronic mucocutaneous candidiasis (CMC) occurs with persistent or recurrent infections with *Candida* spp. in the skin, nails, oral, or genital mucosa; approximately half of all cases studied and genetically characterized worldwide are due to GOF *STAT1* mutations, and the other half are due to mutations in other genes that impair the IL-17-mediated immune response, such as *STAT3, IL17RA, IL17F, IL17RA, IL17RC, ACT1, RORC, IL12RB1, AIRE*, and *CARD9* (17–23).

In 2016, an international multicenter study (24) examined 274 patients with GOF mutations in *STAT1*; while 268 patients had CMC only 17 patients had mycobacterial infections (6% of the studied patients). This is paradoxical and unexplained, as LOF mutations in *STAT1* are well-known causes of mycobacterial disease (25).

## Discussion

We identified a GOF mutation in *STAT1* in our patient with confirmed *M. tuberculosis* disseminated infection and CMC. The mutation found in the patient increased the phosphorylation of Y701 in the STAT1 protein, which was proved to be resistant to Staurosporine, but sensitive to Ruxolitinib, which has previously been used against CMC in two clinically distinct patients (5, 6). This suggests an alternative treatment for infections in this type of patient; however, its effects in the elimination of mycobacterial infections remain to be determined.

Chronic mucocutaneous candidiasis is the most frequent infection associated to *STAT1* GOF mutations (1, 16), although patients have also bacterial and viral infections (24). Interestingly, Sampaio et al. (16) identified a patient with the mutation T385M (the same found in our patient) and a sporadic infection with *Mycobacterium fortuitum*, indicating that there is also susceptibility to Mycobacterial infection with the *STAT1* GOF mutation. The mycobacterial infections found in 17 out of 268 patients with heterozygous *STAT1* GOF mutations recently reported by Toubiana et al. demonstrate the susceptibility to these infections (24).

In the patient reported here, BCG vaccination caused an initial mycobacterial infection during her first year of life and debridement of the axillar abscess relieved infection symptoms. Although AFB were found in gastric juices from patient at 3 years old, isolation of mycobacteria was unsuccessful. Later, the infection in the clavicule showed AFB in the bone and soft tissue and the Gene-X-pert-positive result demonstrated an *M. tuberculosis* infection, with clinical symptoms of dissemination (see Figure 1). Thus, clinical phenotype and laboratory testing indicated that the patient had a *bona fide* disseminated mycobacterial infection. Recently, Baris et al. (26) observed a pulmonary *M. tuberculosis* infection in a patient with the same T385M GOF mutation in STAT1, while Kataoka et al. (27) identified a patient with an R274G *STAT1* GOF mutation and disseminated *M. tuberculosis* infection albeit with negative Quantiferon and tuberculin skin tests. Similarly, PPD testing was negative in our patient at 3 and 6 years old, despite her mycobacterial infections, indicating a defective T-cell response.

The mechanism underlying *STAT1* GOF mutations and susceptibility to mycobacterial infection is not well understood, but cytokine production is altered in leukocytes. Sampaio et al. (16) found increased production of IL-12 as well as chemokines CXCL9 and CXCL10 in response to IFN-γ in patients with *STAT1* GOF mutations. Similarly, we found an increased production of IL-12 (shown) and CXCL10 (not shown) in whole blood from the patient stimulated with BCG compared to healthy controls. IFN-γ is thought to be an essential cytokine necessary to control of mycobacterial infections. However, we found by ELISA only 35% less IFN-γ production in whole blood from the patient stimulated with BCG compared to healthy controls; by flow cytometry, we observed diminished IFN-γ producing T cells in PBMCs from the patient compared to control (29 vs 42%) but almost absent in IL-17A-producing T cells in the patient compared to control (0.2 vs 1.6%), upon PMA + ionomycin stimulation. Liu et al. (1) found a diminished (albeit not significant) percentage of IFN-γ-producing cells in patients with *STAT1* GOF mutations compared to healthy controls and significant lower proportion of IL-17-producing T cells (by flow cytometry) similar to other authors (2, 16, 28). Therefore, consistently, there is a significant reduction in IL-17 production but only partial IFN-γ reduction in patients with *STAT1* GOF mutations. In a murine model of lung infection with BCG, IL-17-deficient mice showed impaired Th1-IFN-γ production and defective granuloma formation compared to wild-type mice (29), suggesting a protective role of IL-17 *in vivo*. However, human patients lacking the IL-17A receptor do not suffer from mycobacterial infections (20), whereas patients with mutations in *RORC* lack the Th1* subset able to produce both IFN-γ and IL-17 (patients do not produce IL-17 A and IL-17 F) and suffer from both *Mycobacterium* and *Candida* infections (23). Mycobacteria induce both Th1 and Th17 responses. T cells from children vaccinated with BCG stimulated *in vitro* with PPD produce high amounts of both IFN-γ and IL-17 compared to non-vaccinated children [reviewed in Ref. (30)]. Interestingly, IL-17 production is reduced in patients with active tuberculosis and its production increases when tuberculosis symptoms are ameliorated after drug treatment, to levels found in healthy controls (31–33), suggesting that IL-17 may participate for the control of mycobacterial infections. Nevertheless, the role of Th17 cells in immune responses against tuberculosis is controversial because there appears to be distinct roles in latent and active human infections, while in animal models, its effect can be beneficial or detrimental (34). Other studies suggest that a high production of Type I IFNs and IL-10 are detrimental during active tuberculosis, inhibiting the protective effects of IFN-γ (35–37). IFN-α has demonstrated beneficial effects as adjuvant during pharmacological treatment of tuberculosis in humans, but paradoxically hepatitis treatment with IFN-α reactivates latent tuberculosis (34). In contrast, Uzel and Holland (38) found a diminished production of IL-10 in response to PHA in PBMC from three patients with STAT1 GOF mutations compared to healthy controls, suggesting that an increased production of IL-10 may not occur in patients with these mutations. The pathway of type I IFNs production and the consequent activated genes has not been studied in patients with *STAT1* GOF mutations and mycobacterial infections and need to be addressed in further studies.

## Concluding Remarks

We described here a patient with a *bona fide M. tuberculosis* disseminated infection, related to a GOF mutation in *STAT1*, a genetic change associated primarily with CMC. GOF mutations in *STAT1* affect IL-17 production, and IFN-γ to a lesser extent. More research is required to investigate the mechanisms associated with IFN-γ and IL-17 cytokine production, distinct types of *STAT1* mutations, and the resulting susceptibility to different infections.

## Materials and Methods

### Laboratory Tests to Identify the Candidate Gene Causing the Immunodeficiency

Several previously published tests (39) were performed to study the function of elements of the IFN-γ/IL-12 circuit. IFN-γ production and function of IL-12 receptor were tested stimulating diluted whole blood with BCG or BCG + IL-12. IFN-γ levels were determined in 48 h supernatants by in-house sandwich ELISA. IL-12 receptor expression was determined by flow cytometry on 3-day PHA-T blasts.

IL-12p70 levels were assessed by ELISA (DuoSet reagents; R&D) in 48 h supernatants of diluted whole blood stimulated with BCG or BCG+ 50, 100, 500, and 1,000 IU of rh-IFN-γ (R&D Minneapolis, MN, USA). IFN-γ receptor 1 expression on CD14+ cells was assessed in fresh monocytes by flow cytometry with anti-CD119-PE (E-Biosciences—Thermo Fisher Scientific, Waltham, MA, USA). All the tests were run with samples from the patient, parents, and volunteer healthy controls.

### Detection of STAT1 and Phospho-STAT1 by Western Blotting

Epstein-Barr virus lymphoblastoid cell lines were maintained in RPMI 1640 supplemented with 10% FBS and antibiotics at 37°C and 5% CO_2_. One day before the experiment, the cells (EBV-LCLs or PBMCs in some experiments) were starved in RPMI with 1% of FBS; the next day, 5 × 10^6^ cells for each condition were washed and placed in 2 mL tubes in serum-free RPMI media and incubated for 2 h at 37°C and 5% CO_2_. Cells were then stimulated with 100 or 1,000 IU IFN-γ or 25,000 IU IFN-α for 30 min. For the experiments with the protein-kinase inhibitor staurosporine, the cells were incubated in RPMI with 2.5% FBS with 1,000 IU IFN-γ for 30 min, then 1 µM of staurosporine was added for 15, 30, and 60 min. After treatments, 1 mL of cold PBS was added to each tube, cells were centrifugated at 17,000 × *g* for 1 min at 4°C and cytoplasmic and nuclear extracts were obtained using a membrane lysis buffer (10 mM HEPES, pH 7.9, 10 mM KCl, 0.1 mM EDTA, 0.1 mM EGTA, 0.05% NP-40, 25 mM NaF, 1 mM Na_3_VO_4_, 1 mM PMSF) and nuclear lysis buffer (20 mM HEPES, pH 7.9, 0.4 M NaCl, 1 mM EDTA, 1 mM EGTA, 25% glycerol, 1 mM PMSF, 25 mM NaF, 1 mM Na_3_VO_4_). Proteins were subjected to SDS-PAGE and transferred to a polyvinylidene difluoride membrane (Millipore, Merck, Billerica, MA, USA). Immunoblotting was performed with anti-phospho-STAT1 Y701 (Cell Signaling), anti-STAT1, and anti-lamin B (Santa Cruz, Dallas, TX, USA) and detected with horseradish peroxidase (HRP)-conjugated goat anti-rabbit IgG (Thermo Scientific). SuperSignal (Thermo Fisher) was used as chemical substrate for HRP.

### Detection of Phospho-STAT1 by Flow Cytometry

Phosphorylation of STAT1 after IFN-γ stimulation was assessed in fresh PBMC from tested subjects, by flow cytometry (40). Briefly, 1 × 10^6^ PBMCs were first membrane-labeled with anti-CD14-FITC (Becton Dickinson, BD, San Jose, CA), washed, stimulated with 1,000 IU of recombinant human IFN-γ or with 50,000 IU of IFN-α (R&D) for 20 min at 37°C in serum-free RPMI. Phosphorylation was stopped by adding 0.5 mL of 4% paraformaldehyde in PBS for 10 min. Permeabilization was performed with 1 mL of 90% methanol + 10% FACSPerm II solution (BD) for 30 min, and intracellular labeling was made with anti-p-STAT1–PE antibody (BD) for 30 min at 4°C. Cells were washed and resuspended in PBS-1% paraformaldehyde for FACS analysis in a BD FACScalibur flow cytometer.

### Inhibition of STAT1 Phosphorylation by Ruxolitinib

Ruxolitinib inhibits JAK1 and JAK2 phosphorylation and, consequently, STAT1 phosphorylation (41). We tested *in vitro* the effect of Ruxolitinib on STAT1 phosphorylation upon IFN-α and γ stimulation. The day before the experiments, EBV-LCLs from the patient and healthy controls were placed in culture in RPMI-2.5% FBS; the next day, cells were washed, placed in 15 mL Falcon tubes at 5 × 10^6^ cells in serum-free RPMI, and were incubated for 2 h at 37°C and 5% CO_2_. Then, cells in tubes were placed in a water bath at 37°C and were incubated with 1 µM of Ruxolitinib (*Invivo* gen, San Diego, CA, USA) for 5 min and then immediately were stimulated with 1,000 IU of human recombinant IFN-γ or 25,000 IU IFN-α for 20 min. p-STAT1 was then detected by Western blot and flow cytometry.

## Ethics Statement

This study was performed in accordance with the recommendations of the committee of ethics in research of the Instituto Nacional de Ciencias Médicas y Nutrición Salvador Zubiran (INCMNSZ). All participants and parents of the patient gave written informed consent in accordance with the Declaration of Helsinki. These studies were approved by the Institutional Review Board of INCMNSZ, protocol code BQO-751-12-16-1.

## Author Contributions

SPS designed the experiments, performed flow cytometry experiments and wrote the paper. JLLF provided medical care to the patient, contributed to medical figures and wrote the clinical description of the case. YG performed the experiments (flow cytometry and sequencing) and collaborated on writing of the paper. LMR and MLVA maintained cell cultures, performed ELISA and Western blot experiments, contributed to figures and participated in manuscript writing. SSP provided the pathology reports and contribute to the pathology figures. MNF and SME provided medical attention to the patient and contributed to the paper writing. JLC, AP and SBD collaborated in the design of the studies, sequencing to determine the mutation and participated in writing the paper MT coordinated the work, edited the manuscript and wrote the paper.

## Conflict of Interest Statement

The authors declare that the research was conducted in the absence of any commercial or financial relationships that could be construed as a potential conflict of interest.
