# Supplementary material for: Disseminated Tuberculosis and Chronic Mucocutaneous Candidiasis in a Patient with a Gain-of-Function Mutation in Signal Transduction and Activator of Transcription 1
Source: Front Immunol. 2017 Dec 6;8:1651. doi: 10.3389/fimmu.2017.01651 (PMC5723642; doi:10.3389/fimmu.2017.01651)
Supplement: Supplementary file 1 [file Image_1.PDF]

1 2 3 4 5 1 2 3 4 5

Cytoplasmic extract

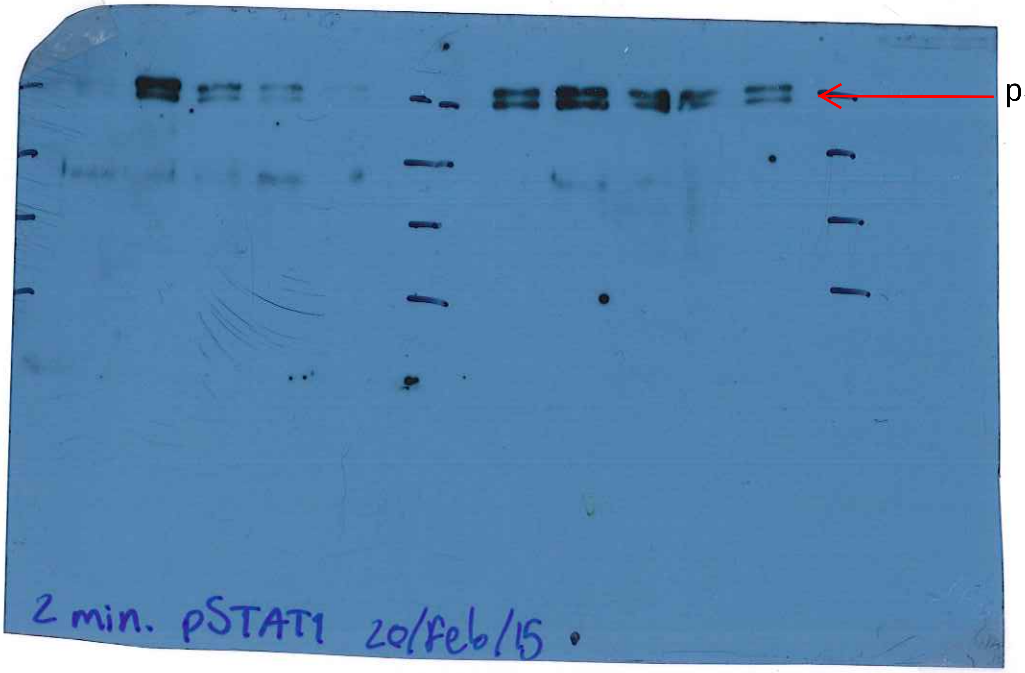

pSTAT-1

1=Non stimulated
2=IFNg 30 min
3=IFNg 30 min + stau* 15 min
4=IFNg 30 min + stau 30 min
5=IFNg 30 min + stau 60 min

HEALTHY CONTROL PATIENT

* stau = staurosporine

1 2 3 4 5 1 2 3 4 5

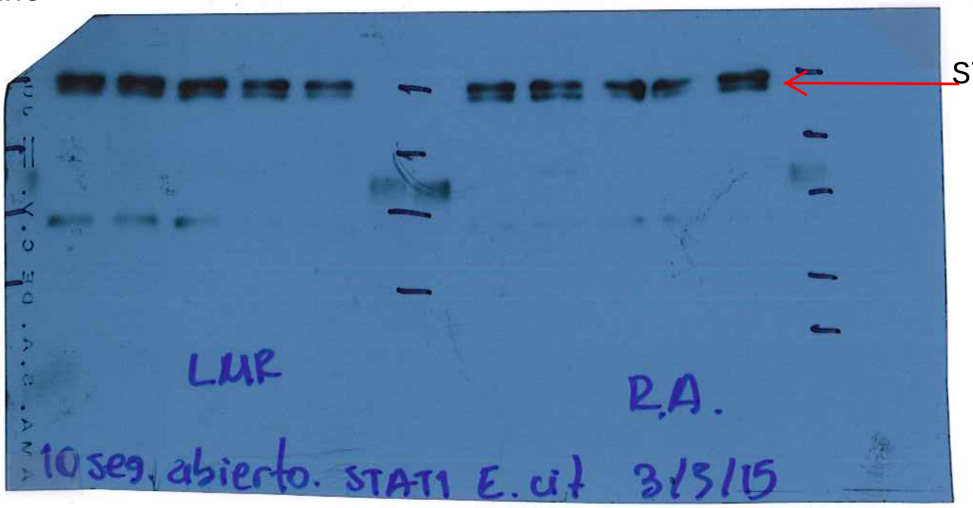

STAT-1

HEALTHY CONTROL PATIENT

1 2 3 4 5 1 2 3 4 5

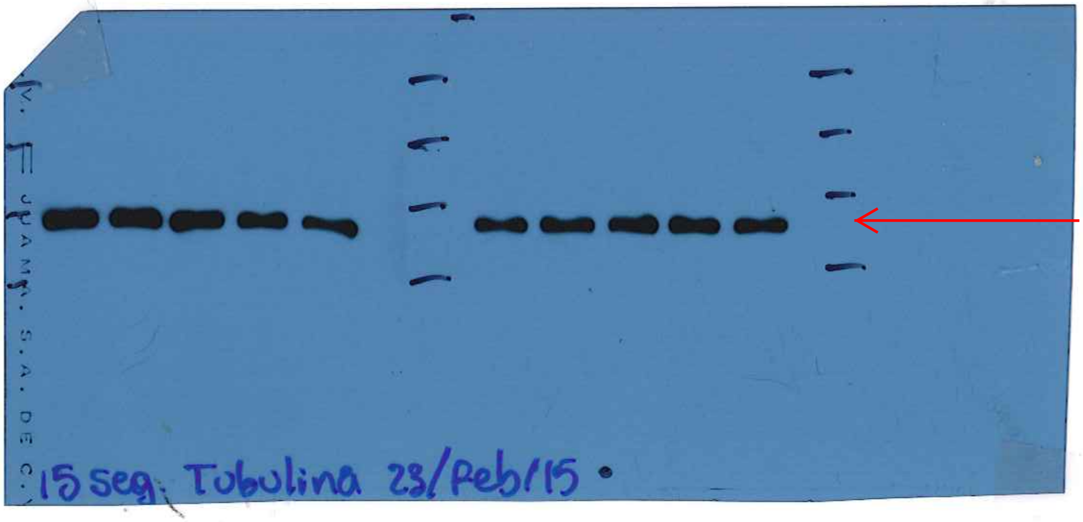

Tubulin

Nuclear extracts

HEALTHY CONTROL PATIENT

1 2 3 4 5 1 2 3 4 5

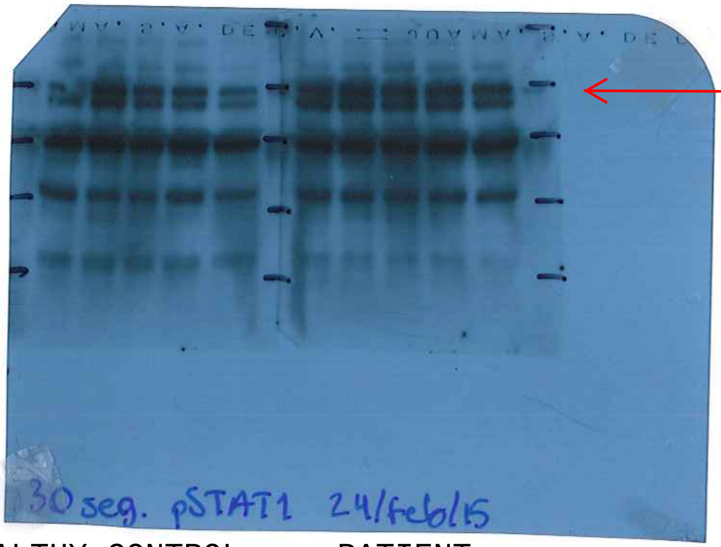

p-STAT-1

HEALTHY CONTROL PATIENT

1 2 3 4 5 1 2 3 4 5

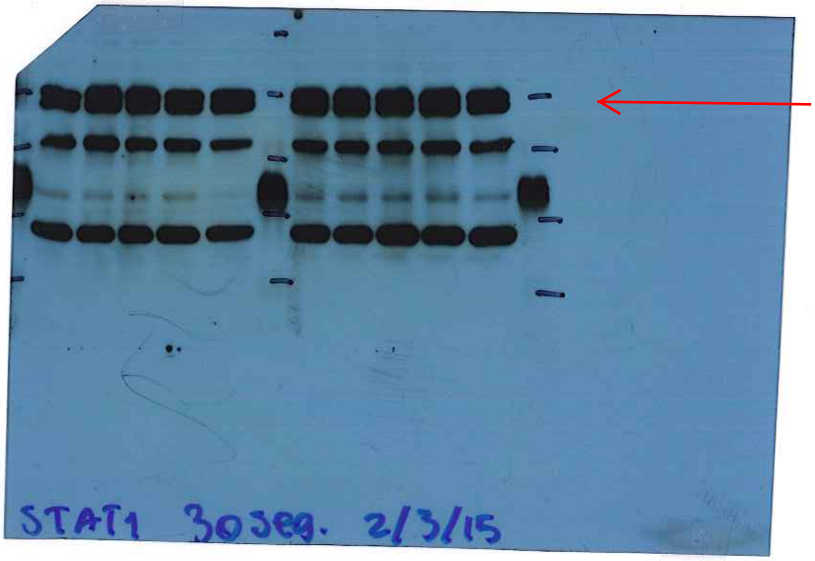

STAT-1

1=Non stimulated
2=IFNg 30 min
3=IFNg 30 min + stau 15 min
4=IFNg 30 min + stau 30 min
5=IFNg 30 min + stau 60 min

1 2 3 4 5 1 2 3 4 5

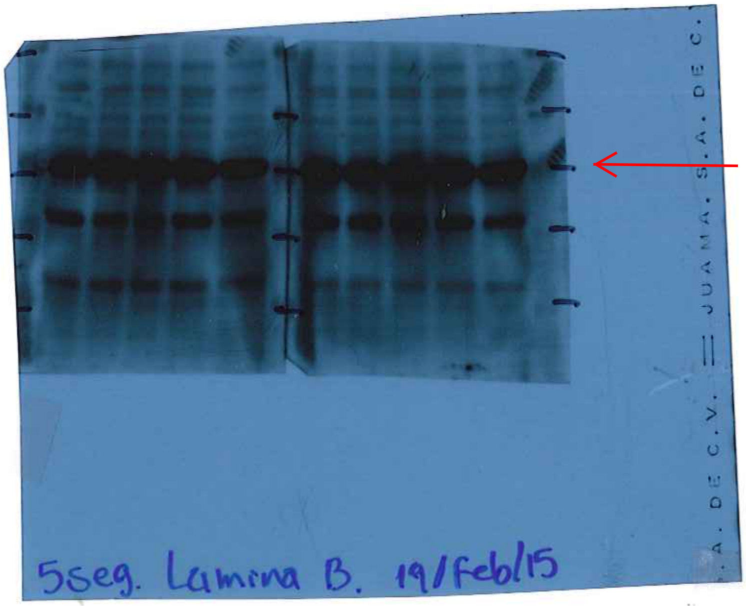

Lamin B

HEALTHY CONTROL PATIENT
